# Three-Dimensional Screen Printing Technology Enables Sequential Release of Carbidopa and Levodopa—A New Approach Improving Levodopa Delivery for Treating Parkinson’s Disease

**DOI:** 10.3390/pharmaceutics17121507

**Published:** 2025-11-22

**Authors:** Marcel Enke, Moritz Bünger, Emily Aedtner, Stephan Kastner, Franka Gruschwitz, Klaus Kühne, Dominika Czernik-Schulz, David R. Greeley, Dieter Volc, Andrea Buzachnich-Ladinig, Achim Schneeberger

**Affiliations:** 1 Laxxon Medical GmbH, Otto-Schott-Str. 15, 07745 Jena, Germany; marcel.enke@laxxonmedical.com (M.E.);; 2 Clinical Centre for Population Medicine in Fish, Pigs and Poultry, University of Veterinary Medicine, Veterinaerplatz 1, 1210 Vienna, Austriaandrea.ladinig@vetmeduni.ac.at (A.B.-L.); 3 Laxxon Medical Corp, 261 Madison Ave, New York, NY 10016, USA; 4 SE Tylose GmbH, Kasteler Str. 45, 65203 Wiesbaden, Germany; 5 Northwest Neurological, PLLC, 1520 W 3rd Avenue, Spokane, WA 99201, USA; 6 Atomos Klinik Waehring, Kreuzgasse 18–19, 1180 Vienna, Austria

**Keywords:** 3D printing, additive manufacturing, levodopa, Parkinson’s disease, oral dosage forms, drug delivery systems

## Abstract

**Introduction:** Levodopa (LD) is the most efficacious antiparkinsonian drug. However, long-term conventional LD treatment of Parkinson’s disease (PD) is frequently associated with motor complications. This can be attributed to pulsatile dopaminergic stimulation given the short LD half-life of conventional dosage forms. Tablets capable of delivering more stable and sustained dopaminergic stimulation would better mimic the brain’s natural dopamine activity. **Methods:** In this study, 3D screen printing technology was used to manufacture oral dosage forms characterized by the sequential release of Carbidopa and Levodopa. This was achieved by separating the two compounds into different compartments within the same dosage form, which were arranged (LXM.5-1) or formulated (LXM.5-2) in a specific way. Both novel dosage forms were compared to conventional immediate release forms such as Sinemet^®^. The physicochemical properties of the resulting tablets, LXM.5-1 and LXM.5-2, were assessed in accordance with the USP. Their pharmacokinetic profiles were defined in pigs. **Results:** The physicochemical properties of LXM.5-1 and LXM.5-2 complied with regulatory requirements. Dissolution studies revealed sequential CD and LD release for both novel dosage forms. They differed regarding the interval between CD and LD release which was shorter for LXM.5-1. PK studies demonstrated that both novel dosage forms exhibited higher LD bioavailability in comparison to Sinemet^®^, which was 211.36% and 383.64% for LXM.5-1 and LXM.5-2, respectively. Furthermore, blood levels were more stable and sustained, particularly for LXM.5-2. **Conclusions:** We conclude that 3D screen-printed LXM.5-1 and LXM.5-2 and variations thereof have the potential to transform the pharmacotherapy of Parkinson’s disease.

## 1. Introduction

Parkinson’s disease (PD) is the second most prevalent neurodegenerative disorder, impacting approximately 0.3% of the population in Western countries. Its prevalence escalates with age, affecting about 1% of individuals aged 60 and reaching around 4% by age 80 [1]. It is estimated that PD cases will double within the next two decades [2,3].

PD is a progressive condition marked by a variety of motor and non-motor symptoms (NMSs). Its pathological hallmark is the accumulation of misfolded, fibrillar alpha-synuclein (aSyn) in neurons. Key motor symptoms include bradykinesia, rigidity, and resting tremor. NMSs also significantly impact those with PD. These include sleep disturbances, dysphagia, constipation, apathy, depression, pain, and autonomic dysfunctions, which collectively can severely affect the quality of life of patients and lead to disease-related complications. The severity of these symptoms often dictates the overall impact of the disease on the individual [4]. The clinical presentation and progression of PD can vary greatly among patients [5].

Levodopa (LD) is the most potent antiparkinsonian drug available. Ever since pioneering reports in the 1960s, it has represented the gold standard for the treatment of PD. It increases dopamine levels in the brain, alleviating motor symptoms like bradykinesia and rigidity. LD is combined with either a decarboxylase inhibitor (Carbidopa (CD), Benserazide) or a COMT inhibitor (Entacapone, Tolcapone) to prevent its peripheral degradation (after crossing the blood–brain barrier, Levodopa is transformed into dopamine by cerebral decarboxylases). These agents increase LD’s bioavailability and duration of action. Fixed combinations include LD/CD (added to the WHO list of essential medicines in 1977), LD/Benserazide, and LD/CD/Entacapone. Newer formulations such as Rytary^®^ and Crexont^®^ combine immediate-release (IR) LD/CD and extended-release (ER) LD/CD-containing beads (Rytary^®^) or ER LD pellets (Crexont^®^) in capsules. This keeps LD blood levels steady for longer periods of time. However, the LD bioavailability of Rytary^®^ (70–80%) and Crexont^®^ (88–99%) is reduced compared with IR LD/CD preparations (e.g., Sinemet^®^) necessitating higher doses. Of note, both the conventional and these newer LD dosage forms result in the concomitant release of LD and the decarboxylase inhibitor. To the best of our knowledge, there is currently no LD-containing dosage form available that would have been produced using additive manufacturing.

Despite its effectiveness, the chronic use of conventional oral Levodopa formulations is associated with significant long-term motor complications, notably motor fluctuations and dyskinesias. Within 5 years of conventional LD treatment, 40–50% of patients develop dyskinesias and motor fluctuations, increasing to 70–80% after 10 years [6]. Furthermore, LD’s effectiveness declines in advanced PD due to fluctuating medication periods and its short half-life. Patients often need to increase the dose and frequency of dosing, which in turn increases the incidence of dyskinesias [7]. These adverse effects are strongly correlated with pulsatile dopaminergic stimulation resulting from the pharmacokinetic profile of immediate-release levodopa, which leads to non-physiological oscillations in LD plasma and synaptic dopamine concentrations. They contribute to a 41% increase in direct medical costs [8].

Emerging evidence from both clinical and preclinical studies supports the hypothesis that the pattern of dopaminergic delivery, rather than the dose alone, plays a critical role in the development of these complications [9]. Non-pulsatile (continuous or sustained) dopaminergic stimulation (CDS) aims to more closely approximate endogenous dopaminergic neurotransmission by maintaining relatively stable plasma Levodopa levels and thereby achieving more uniform receptor activation in the striatum. Therapeutic strategies designed to implement CDS include Levodopa–Carbidopa intestinal gel (LCIG) infusion [10], controlled-release oral formulations, and the adjunctive use of enzyme inhibitors (e.g., COMT and MAO-B inhibitors) to extend the half-life of Levodopa. These approaches have demonstrated efficacy in reducing both motor fluctuations and dyskinesias, supporting the pathophysiological rationale underlying CDS [10].

Currently, 3D screen printing is a cutting-edge additive manufacturing technology [11,12]. It enables the production of pharmaceutical dosage forms of unprecedented complexity in large quantities. A single production run can produce units of different shapes and sizes [12]. The release of the active ingredient can be precisely controlled by defining the internal architecture, the size and geometry of the units, and the carrier materials used. As the 3D screen printing process does not involve high temperatures or significant pressure, it can be used with a wide range of substrates and drug substances.

Unlike other manufacturing technologies, 3D screen printing offers the possibility of separating drugs into different compartments of a given oral dosage form. This has the potential to differentially regulate the release of the different drugs. This is of particular interest to oral LD-containing dosage forms, as LD is combined with CD to increase its bioavailability. We hypothesized that the bioavailability of LD could be increased further if the CD were released before the LD, based on its inhibitory action against LD degradation. To this end, we harnessed the potential of additive manufacturing to design two novel dosage forms containing CD and LD in different compartments. Two principal strategies were followed, namely, controlled drug release by the architecture of the dosage form and controlled drug release as a function of the composition of the different compartments. In LXM.5-1 (LXM.5 is an internal project code referring to Laxxon’s novel LD/CD dosage forms; LXM.5-1/2 refers to specific prototypes as described in the manuscript), the release of both drugs was tailored through the geometry of the dosage form whereas in LXM.5-2 the release of the two drugs was controlled by the composition of the compartments, a key component being a pH-sensitive polymer built into the LD compartment. Their evaluation confirmed the concept of increasing the bioavailability of LD by sequentially releasing CD and LD, with CD preceding LD. Furthermore, not only were LD blood levels higher, but they also persisted for longer. These studies have several potential implications which range from practical consequences, such as reducing the number of daily intakes, to the potential to slow the progression of the disease.

## 2. Materials and Methods

### 2.1. Chemicals

CD and LD were purchased from Divi’s Laboratories, Hyderabad, India. The various excipients were purchased from established providers: Klucel LF (Ashland, Covington, KY, USA), Avicel PH 105 (Dupont, Wilmington, DE, USA), starch 1500 (Colorcon, Harleysville, PA, USA), glycerol and triactin (AppliChem, Darmstadt, Germany), talc (Imerys, Paris, France), Silfar 350 and Silfar SE4 (Wacker Chemie, Munich, Germany), L-ascorbic acid and aqueous HCl (Carl Roth GmbH, Karlsruhe, Germany), citric acid (Honeywell Fluka^TM^, Charlotte, NC, USA), Ac-Di-Sol (IFF, Roquette, Lestrem, France), Mannogem Emerald (SPI Pharma, Wilmington, DE, USA), and Miglyol 812 N (IOI Oleo, Hamburg, Germany). Some materials were kindly provided by the respective manufacturers. This includes Shin-Etsu AQOAT^®^ AS-LF provided by SE Tylose GmbH & Co.KG, Wiesbaden, Germany and RxCIPIENTS^®^ FM 1000 by Evonik, Darmstadt, Germany. Demineralized water was used for all formulations and solutions. For HPLC measurements, sodium dihydrogen phosphate and HPLC-grade solvent Chemsolute (water and acetonitrile) from TH.Geyer, Renningen, Germany were used.

### 2.2. Preparation of Printing Pastes

All pastes were prepared using an HRV-S 2DP vacuum dissolver planetary mixer with 3 agitator tools from Herbst Maschinenfabrik GmbH (Buxtehude, Germany), in a 2-L glass container.

The pastes were prepared according to the compositions in Table 1 and Table 2 at room temperature. Briefly, the binder solution (Klucel LF, 10%) was prepared. The appropriate amount of binder gel was then added into the 2-L glass container of the planetary mixer. Then half of the desired water was added. In the next step, all solid excipients were added starting with the highest in mass. Subsequently, all liquid excipients were added. Before mixing, the API and the residual water were added. The final paste was mixed for 20 min at 1000 rpm under 70 mbar vacuum.

### 2.3. Rheology Measurements

Rheology measurements of paste P1 to P4 were performed on a Kinexus Rheometer (Netzsch, Selb, Germany) equipped with a plate–plate geometry and a passive solvent trap at 25 °C. A 500 µm gap was selected. In the 3-step shear rate test, shear rates of 0.1 s^−1^ (for 1.5 min), 100 s^−1^ (for 0.5 min), and 0.1 s^−1^ (for 10 min) were applied. The amplitude sweep was conducted at 1 Hz, and the frequency sweep at 0.05% strain.

### 2.4. Three-Dimensional Screen Printing of Tablets

LXM.5-1 and LXM.5-2 were manufactured on a prototype lab-scale 3D screen printing unit, XHS STS 3D (Exentis Group AG, Stetten, Switzerland), in accordance with the previously delineated methodology [12]. Printer settings were as follows: flooding and printing squeegee speed 100 mm/s, off-contact distance 2 mm, height increment for screen elevation 15 µm, dry power 65%, and drying time per layer 15 s.

### 2.5. Tablet Hardness and Friability Testing

The fracture resistance of tablets was detected using tablet hardness testing instrument PTB 51 1E by Pharma Test (Apparatebau AG, Hainburg, Germany), according to Ph. Eur. 2.9.8. The average and standard deviation of 5 randomly selected tablets per batch were calculated.

Friability was tested as described in Ph. Eur. 2.9.7. Deviating from the method, 5 g tablets of LXM.5-2 and 5 g tablets of LXM.5-1 were dedusted by airflow and weighed, then rotated 100 times at 25 rpm on a single drum tablet friability test instrument PTF 100 by Pharma Test (Apparatebau AG, Hainburg, Germany). Friability was determined as the percentage of weight loss after a further dedusting of the tablets and should be less than 1%.

### 2.6. Mass Uniformity

Mass uniformity, a pharmacopeial requirement (see Ph. Eur. 2.9.5), was determined by means of weighing 20 tablets from each batch, followed by the calculation of the means and standard deviations. Semi-micro balance Sartorius^TM^ Secura 125-1S G2324665 (Sartorius, Göttingen, Germany) was used.

### 2.7. Dimension Uniformity

In order to ascertain the uniformity of the dimensions of the tablets, a total of 20 tablets from each formulation were meticulously measured (diameter, height) using a Digimatic Caliper CD-15APX by Mitutoyo, Kawasaki, Japan (certified). The mean values and standard deviations for tablet dimensions and volumes were then calculated.

### 2.8. Disintegration Testing

Disintegration was tested according to Ph. Eur. 2.9.1 with a Pharmatest Auto1EZ by Pharma Test Apparatebau AG, Hainburg, Germany, an automatic disintegration time tester using disks. Demineralized water (750 mL, 37 °C) was used as test media. The mean value and standard deviation were subsequently calculated.

### 2.9. In Vitro Dissolution Testing

The in vitro dissolution test was performed according to USP <711> in a paddle apparatus (USP apparatus 2) in 900 mL 0.1 M HCl. The number of each type of tablet tested in the dissolution apparatus was six tablets. However, only two tablets of the Sinemet tablet were investigated because it is a well-known tablet on the market. As the solubility products for levodopa and carbidopa are 5000 mg/l and 3.8 mg/L, respectively [13], to achieve sink conditions, concentrations V/V_sat_ ≥ 3 [14] were used in the vessel being 100 mg/900 mL for Levodopa and 25 mg/900mL. To comply with USP43-NF38-753 dissolution test 1 the rotation speed was set to 75 rpm for the dissolution tests in formulation development Testing the influence of stirring speed by varying it between 50, 75, 100, and 150 rpm revealed that there was no further influence on the dissolution beyond 100 rpm. Thus, this speed was chosen for further experiments.

For LXM.5-2 the dissolution method was extended due to the prolonged release. Therefore, Method A of the chapter Extended-Release Dosage Forms in USP <711> were utilized.

### 2.10. High Performance Liquid Chromatography (HPLC) for LXM.5-1 and LXM.5-2

The liquid chromatograph was equipped with a Diode Array Detector detector adjusted to 280 nm and a 150 mm × 4.6 mm 5 µm ODS C-18 column (Agilent Zorbax Eclipse Plus C18 by Agilent Technologies, Santa Clara, CA, USA) at 40 °C. Injection volume: 5 µL.

Procedure: Protect all solutions from sunlight and maintain them at 10 °C until they are injected into the chromatograph.

Mobile phase: 50 mM NaH_2_PO_4_; adjust with Phosphoric acid and Acetonitrile to a pH of 2.7 in 95:5 ratio.

Stock solution: 1 mg/mL of Levodopa in 0.1 M HCl, 1 mg/mL of Carbidopa in 0.1 M HCl.

Standard solution 100 µg/mL of LEV and 30 µg/mL CAR in 0.1 M HCl.

Calibration solution: 100, 50, 25, 12.5, and 6.25 µg/mL in 0.1 M HCl.

Sample solution: 1 mL of the dissolution test solution at the specific time point.

The method was not validated but is in accordance with the USP method [15].

### 2.11. In Vivo Pig Study

The pharmacokinetic study was achieved in collaboration with the University of Veterinary Medicine Vienna, University Clinic for Swine, Vienna, Austria, for the in vivo part. The allowance of the study was granted by the respective Animal Welfare Committee (GZ: 2023-0.529.291 Approved on 23 July 2023). For the application of the tablet using a gastroscope (Olympus, Shinjuku, Japan), the study animals (n = 4/group) fasted overnight and were anesthetized (0.5 mg/kg bw azaperone and 10 mg/kg bw ketamine intravenously or 2 mg/kg bw azaperone and 25 mg/kg bw intramuscularly). To determine the pharmacokinetic profile of the levodopa/carbidopa dosage forms, blood samples (maximum blood volume per time point: 2 mL) were taken shortly before application of the tablets (time point 0 h) and after application of the tablet (30, 60, 90, 180, 240, 360, 480 min, and 24 h) by puncturing the vena cava cranialis or vena jugularis with 21G needles. Plasma samples were frozen and stored at −80 °C until analysis. Plasma concentrations of carbidopa and levodopa were determined by HPLC at each sampling time. All study animals are euthanized under general anesthesia at the earliest time after the end of the experiment (24 h after application of the tablets). Pigs had to be older than 6 weeks and weigh less than 25 kg. Mean weight was 15.8 ± 2.4 kg.

### 2.12. LS-MS Analytics of Levodopa and Carbidopa in Porcine Plasma

The method from [16] was used. The HPLC system consisted of a Vanquish U-HPLC pump and a Vanquish Autosampler (Thermo Fisher Scientific, Waltham, MA, USA). Mass spectrometry was performed on a Q-Exactive mass spectrometer (Orbitrap^TM^ technology with accurate mass) equipped with an H-ESI (heated electrospray interface) (Thermo Fisher Scientific, Waltham, MA, USA) connected to a PC running the standard software Chromeleon 7.2.10. The quantitative determination of Levodopa and Carbidopa in plasma was carried out by reversed phase HPLC employing High-Resolution Mass Spectrometry (HRMS) as the detection method. All analytical samples were stabilized by adding 4% of a 10% sodium metabisulfite solution to protect the analytes from degradation.

The validated calibration ranges for measuring Levodopa and Carbidopa in porcine plasma were 75–5000 ng/mL and 3–600 ng/mL, respectively. Sample preparation was performed by protein precipitation.

## 3. Results

### 3.1. LXM.5–1 and LXM.5–2: Two Approaches to Sequential Drug Release Realized by 3D Screen Printing—Design of Tablets and Corresponding Pastes

**Figure 1:**
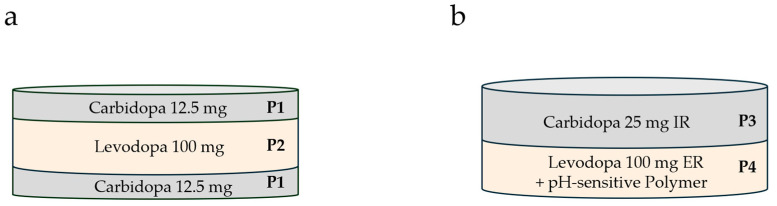
Design of LXM.5-1 (**a**) and LXM.5-2 (**b**), representing the two main approaches to achieving sequential drug release. (**a**) Efficient geometric solution for sequential drug release. All 3 compartments of the sandwich arrangement realized in tablet LXM.5-1are made up of pastes of comparable composition (immediate-release type of paste formulation; see Table 1. The compartment layout maximizes the outer surface area toward the carbidopa compartments which is believed to result in an initial predominance of carbidopa release. (**b**) The formulation solution, realized in LXM.5-2, is based on two compartments differing in their drug release kinetics. Carbidopa is placed in an immediate-release type of environment while levodopa is embedded in an extended-release matrix with a pH-sensitive polymer, focusing LD release to the upper intestine. IR: immediate release, ER: extended release.

Additive manufacturing offers the possibility of the compartmentalization of drugs within oral dosage forms. The presence of different compartments allows us, among other benefits, to tailor the drug release. Here, we tested the feasibility of improving the pharmacokinetics of levodopa by delaying its release compared with its combination partner carbidopa, which is reported to prevent the peripheral degradation of levodopa (thus increasing LD’s bioavailability) [17,18]. In principle, there are two ways to achieve the sequential release of active ingredients (Figure 1). First, by appropriately arranging the compartments with the different active ingredients (solution by geometry; Figure 1a). Second, by separating the two drugs into compartments characterized by different release kinetics, achieved by their composition (solution by paste formulation; Figure 1b). Sequential release in this case means the sustained release of levodopa, while carbidopa is released immediately. Levodopa release starts concomitantly with carbidopa, but with a lower release rate, to gain an initial carbidopa excess (compared with standard levodopa/carbidopa dosage forms) in the blood plasma.

Levodopa has a narrow absorption window in the proximal small intestine (duodenum and jejunum) and thus uptake is dependent on gastric emptying. Absorption through the intestinal mucosa is reported to be mediated by transporter systems (SLC7A9–SLC3A1) with leucine and arginine as competitors. Whereas transport in the central nervous system is mediated by LAAT transporter SLC6A19 [19]. Carbidopa’s absorption pathway is *not* well defined, rather, it is thought to be absorbed by other, non-saturable mechanisms and/or passive diffusion [20]. The literature emphasizes that carbidopa’s bioavailability is not affected by the presence of levodopa, but by food intake [17].

**Figure A1:**
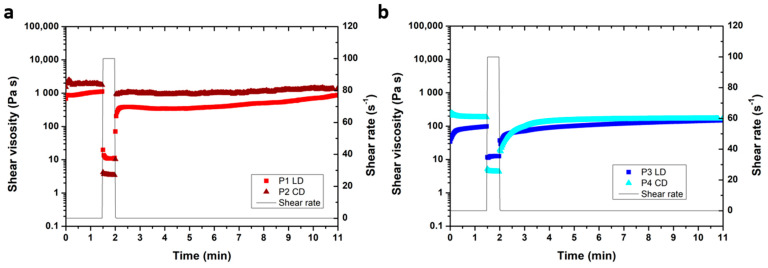
The flow characteristics of the four pastes for LXM.5-1 are assessed via 3-step shear rheology measurements. P1(LD, light red) and P2 (CD, dark red) (**a**), and P3 (LD, dark blue) and P4 (CD, light blue) (**b**) were subjected to shear rates of 0.1 s^−1^ for 1.5 min, 100 s^−1^ for 0.5 min, and 0.1 s^−1^ for 9 min at 25 °C. All pastes show low thixotropic behavior with shear thinning at high shear rates.

*Tablet LXM.5-1.* Concerning the first approach (tailoring drug release through the geometry of the dosage form), we chose a sandwich-type arrangement (Figure 1a). The outer compartments contain Carbidopa, while the middle contains Levodopa. The formulations of the Carbidopa- and Levodopa-containing pastes that make up the three compartments are shown in Table 1. They are identical in their general formulation (types and relative amounts of excipients) and therefore largely in the release kinetics of the respective active ingredients. Based on the type and relative amounts of polymer(s) used, the release kinetics of the compartments were set to immediate release. They obviously differ in the type and amount of active ingredients: 25 mg CD and 100 mg LD (based on the mass of the resulting tablet). Another minor difference is the addition of a small amount of the antioxidant citric acid to P1, the CD paste. The rheological characteristics of both pastes used to build LXM.5-1, the CD paste (P1) and the LD paste (P2), are shown in the Appendix A Figure A1. Both pastes exhibit low thixotropic behavior with immediate shear thinning upon increased shear stress and a fast recovery to initial gel strength upon removal of the shear force. These flow characteristics have been proven to result in good printability such as an even printout and good height buildup [11].

The two CD compartments show an SA/V of 0.68 mm^−1^, whereas the LD compartment has an SA/V ratio of 0.39 mm^−1^ based on the total volume of the tablet. The CD:LD ratio is 1:0.57, thus carbidopa has a higher surface area to the total volume of tab 1 resulting in the faster dissolution of this compartment and thus the faster release of carbidopa. Based on the total surface area, the CD compartment covers 63.42% of the tablet LXM.5-1, while the LD compartment covers 36.58% (see Appendix A Table A1).

**Figure 2:**
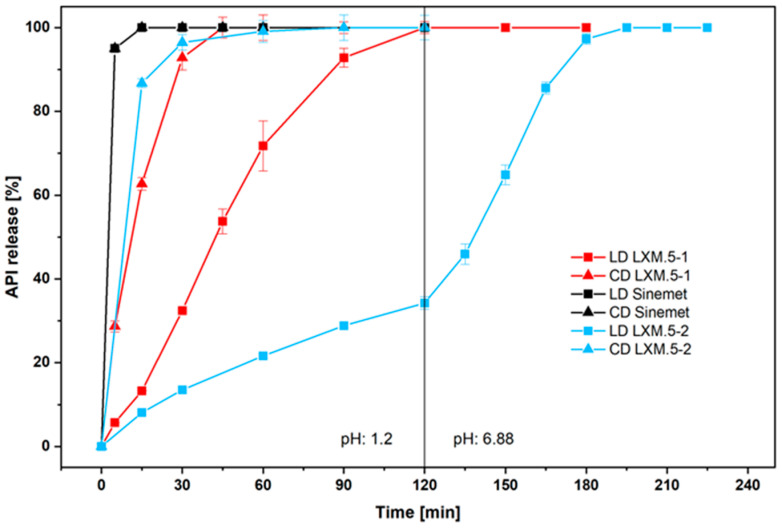
Relative in vitro CD and LD release of LXM.5-1 (red) and LXM.5-2 (blue) compared to conventional immediate release-type Sinemet^®^ tablets. Note: for LXM.5-1 and Sinemet the pH was constant at 1.2 during the experiment; for LXM.5-2 the medium was changed at 120 min from pH 1.2 to 6.8.

*Tablet LXM.5-2.* The second solution for sequential CD and LD release, realized in LXM.5-2 (Figure 1b), is based on the formulations of the two pastes (Table 2) that are used to build the two compartments of the oral dosage form. P3, the CD-containing paste, is designed for immediate drug release. Compared with P1/2, P3 was modified for the even faster release of CD. Specifically, this was achieved by exchanging crospovidone (polyplasdone) with sodium croscarmellose (Ac-Di-Sol). These changes allowed for complete CD release within 30 min (Figure 2). In contrast, P4 was designed to provide the extended release of LD. To this end, a long-chain and pH-sensitive polymer, hydroxy propyl methyl cellulose acetate succinate (Shin-Etsu AQOAT^®^ AS-LF) was chosen as the backbone of the paste. The unique property to incorporate the enteric polymer into the tablet compartment is enabled by the additive manufacturing process. The rheological characteristics of P3 and P4 are shown in the Appendix A. Similarly to P1 and P2, both pastes, P3 and P4, exhibit low thixotropic behavior with immediate shear thinning upon increased shear stress and a fast recovery to initial gel strength upon the removal of the shear force (see Appendix A Figure A1). These flow characteristics also yield good printing characteristics [11].

Note that the surface-area-to-volume ratio of the CD and LD compartments is reversed in LXM.5-2 compared with LXM.5-1. The Carbidopa compartment has an SA/V ratio of 0.41, while the Levodopa compartment has an SA/V ratio of 0.67. The CD:LD ratio is 1:1.68, thus Levodopa has a higher surface area to the total volume of LXM.5-2.

LXM.5-2 was designed so that the Levodopa compartment covers 62.37% of the tablet’s total surface area, while the Carbidopa compartment constitutes only 37.63% (see Appendix A Table A1).

### 3.2. In Vitro Release Profiles of LD and CD in LXM.5-1, LXM.5-2, and the Conventional Dosage Form

**Figure 3:**
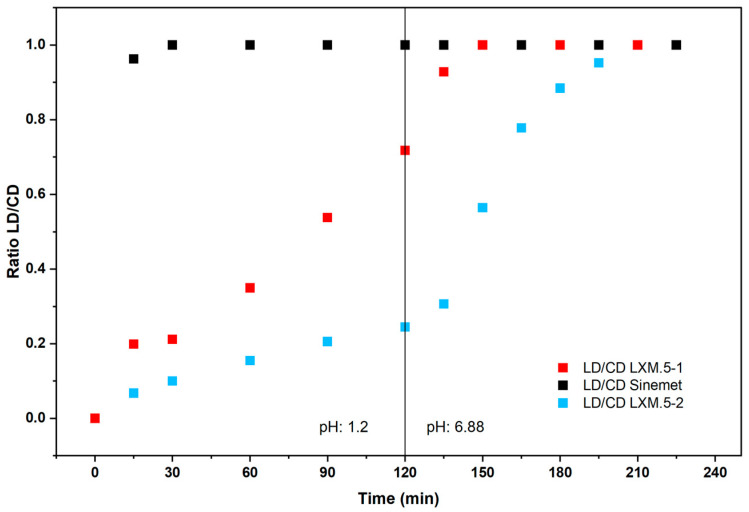
Ratio of LD/CD over time for conventional immediate-release tablet (Sinemet^®^) compared to LXM.5-1 and LXM.5-2.

The disintegration of LXM.5-1 and LXM.5-2 follows the rules of surface erosion [21]. According to this, the compartment with the larger external surface area will release its drug more quickly. It is worth noting that the surfaces covered by the CD compartments were different for LXM.5-1 (63%) and LXM.5-2 (37%). For reference, the conventional, immediate-release CD/LD product Sinemet^®^ [22] was used. In vitro dissolution studies showed that for Sinemet^®^ the relative release curves of CD and LD were superimposed. This is well known from the literature and reflects the homogeneous distribution of CD and LD throughout the conventional dosage (Figure 2). This was different for both LXM.5-1 and LXM.5-2. In both cases, the release of CD was earlier compared with LD (Figure 2; note: while CD release for LXM.5-1 and LXM.5-2 preceded LD release in each case, it was slower compared with CD release of Sinemet^®^). For LXM.5-1, the time interval between the release of 50% of each drug was 30 min. This difference was even more pronounced for LXM.5-2, amounting to 110 min. This longer interval was primarily due to a more delayed release of LD. The kinetics of its release out of LXM.5-2 are further driven by the pH-sensitive release properties of the polymer used for the formulation of the respective compartment, HPMCAS. For the first 120 min, at pH 1.2, only about 35% of the LD was released. Upon increasing the pH to 6.8 the remainder of the LD was fully released within another 60 min. It is important to note that for both LXM.5-1 and LXM.5-2, LD release begins when the tablets are placed into the dissolution vessel. This aligns with their geometry as both dosage forms have external surfaces covering CD compartments. The difference from a conventional LD/CD tablet lies in the ratio of CD to LD release at any time after the dissolution experiment begins. To visualize this, we plotted the CD/LD ratio for the conventional dosage form (Sinemet^®^), as well as for LXM.5-1 and LXM.5-2. It remains at four for conventional LD/CD tablets throughout the study. However, the ratio for LXM.5-1 starts out low and reaches four after 135 min (Figure 3). A similar curve was obtained for LXM.5-2, which did not reach a value of four until 200 min had passed. We are not aware of any published CD/LD dosage forms that would have comparable in vitro release profiles.

Thus, the data obtained is consistent with the rationale behind the design of these novel CD/LD dosage forms. Both LXM.5-1 and LXM.5-2 demonstrate a significantly delayed in vitro release of LD compared with the conventional immediate-release dosage form. To further increase the observed differences in CD and LD release for LXM.5-1, the size of the CD compartment could be enlarged by reducing the concentration of CD and a delayed-release LD paste formulation can be used. The size of the LD compartment could be reduced for LXM.5-2 by increasing the LD concentration of the respective paste. These modifications to the tablet geometry and paste formulation would result in a quantitative change to the drug release profiles of both novel dosage forms. The key advantage of the chosen geometries lies in their ease of manufacturing using 3D screen printing technology. It would also be possible to entirely separate CD and LD release using this technology. To this end, one would require a third compartment that would encase the LD compartment. While this is feasible with 3D screen printing, it would considerably lengthen the manufacturing time.

### 3.3. Characteristics and Appearance of 3D Screen-Printed Tablets LXM.5-1 and LXM.5-2

Following the successful in vitro dissolution testing, the LXM.5-1 and LXM.5-2 tablets were evaluated for their adherence to pharmacopeial standards. Therefore, we tested their physicochemical properties according to European Pharmacopeia (see Section 2.5, Section 2.6, Section 2.7 and Section 2.8).

**Figure 4:**
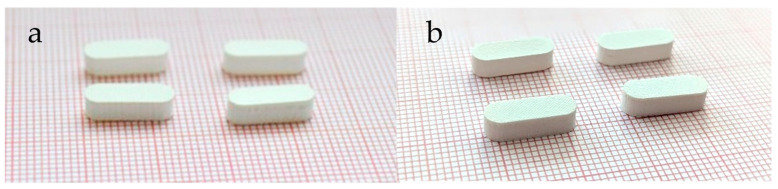
Images of tablets LXM.5-1 (**a**) and LXM.5-2 (**b**) positioned on millimeter paper. Visually, there is no discernible difference between the 3-layer-sandwich tablet LXM.5-1 (**a**) and the 2-layer tablet LXM.5-2 (**b**). The dimensions of the tablets are 14 mm × 5 mm × 3.6 mm (L × W × H).

Both dosage forms, LXM.5-1 and LXM.5-2, were designed in oblong form (Figure 4a,b). They exhibit a uniform white coloration and a coarse surface texture. There is no visual difference between the two. In their larger diameter, both dosage forms were slightly smaller than the specified size defined by the screen layout (Table 3). This phenomenon has been reported before and is likely due to water loss during the drying process [12].

The friability of both dosage forms was significantly lower than 1% and therefore conform with the Eur. Ph. and USP (Table 3). This was also true for the hardness and mass uniformity. LXM.5-2 demonstrated a higher hardness compared with LXM.5-1. Mass uniformity was 1.73% for LXM.5-1 and 0.60% for LXM.5-2. The residual water content was found to be higher for LXM.5-1, though it was within an acceptable range.

### 3.4. Sequential CD and LD Release as Realized with LXM.5–1 and LXM.5–2 Increases LD Bioavailability and Smoothens Its PK Profile

**Figure 5:**
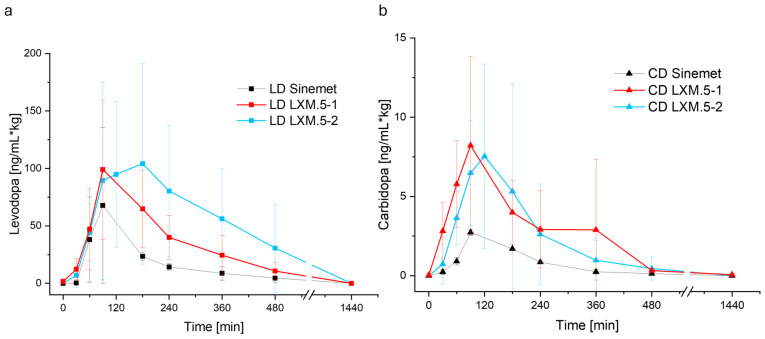
In vivo data derived from pig study of LXM.5-1 (red) and LXM.5-2 (blue). In (**a**) the LD plasma levels and in (**b**) the CD plasma concentrations are displayed. In vivo data of LxM.5-1 shows similar behavior to Sinemet (black) with higher AUC. LXM.5-2 depicts a prolonged levodopa plateau.

We next assessed the PK profiles of LXM.5-1 and LXM.5-2 in pigs. To ensure the integrity of the tablets, they were administered to the stomach of anesthetized animals using a gastroscope. It was expected that the earlier presence of CD would result in higher, prolonged levels of LD in the blood by preventing its degradation in the periphery; the results obtained confirmed this notion. Both LXM.5-1 and LXM.5-2 were found to have a higher bioavailability of LD compared with the conventional, immediate-release formulation, Sinemet^®^ (Figure 5, Table 4). The LD bioavailability for LXM.5-1 was found to be 211.36% and 383.64% for LXM.5-2 compared with Sinemet^®^. A comparison of LD profiles showed that the curves for the first 100 min are nearly identical (see Figure 5; Table 4). The time of the rise in blood levels and the steepness of the curves were comparable between the new formulations, LXM.5-1 and LXM.5-2, and conventional Sinemet^®^. The subsequent course of the curves illustrates the primary distinctions between the novel and traditional dosage forms. Firstly, the LD blood level rise elicited by both new dosage forms is higher than the level seen with Sinemet^®^. Secondly, in contrast to Sinemet^®^, both novel dosage forms were found to exhibit a shoulder in their PK profiles. This was especially apparent with LXM.5-2. The more pronounced shoulder of LXM.5-2 may result from the longer interval between CD and LD release compared with LXM.5-1. Alternatively, it may reflect the steep increase in LD release associated with a pH change (at the transition of the tablet from the stomach to the intestine) based on the use of a pH-sensitive polymer, HPMCAS in LXM.5-2. Finally, the plateau observed in LXM.5-2 may be attributable to both parameters. Although it may seem counterintuitive that LD blood levels rise at the same time and rate as seen with conventional, immediate-release Sinemet^®^, it is important to remember that due to the geometries of LXM.5-1 and LXM.5-2, LD is released immediately after administration, but in much smaller amounts compared with the conventional dosage form. PK profiles, however, revealed another surprising difference between conventional Sinemet^®^ and the new formulations. The two new dosage forms, LXM.5-1 and LXM.5-2, also differ in the significantly higher CD bioavailability to Sinemet^®^. Their relative CD bioavailability was 308.73% and 313.69%, respectively (Table 4). The CD curves reveal several noteworthy findings. Compared with Sinemet^®^, CD blood levels rise more quickly and to a higher level and remain detectable for longer.

## 4. Discussion

We hypothesized that the bioavailability of LD from solid oral dosage forms could be increased through the sequential release of CD and LD, with the decarboxylase inhibitor being released first. To this end, we used 3D screen printing technology to create tablets with different compartments containing either CD or LD. We evaluated two approaches. The first approach (realized in LXM.5-1) was based on the geometry of the tablet to control the drug release. The second approach (LXM.5-2) used different formulations to achieve immediate (for CD) or extended (for LD) drug release. We further tested whether the addition of a pH-sensitive polymer would enhance the bioavailability of LXM.5-2 by tailoring the LD release to the small intestine. Three-dimensional screen-printing technology was found to be well suited to realize both product candidates. The resulting tablets fulfilled the requirements of EU and US pharmacopeia. Both approaches were shown to increase the bioavailability of LD and to extend its blood levels.

Based on current knowledge, the faster and greater increase in the CD blood levels of LXM.5-1 and LXM.5-2 compared with Sinemet^®^ is difficult to reconcile with the in vitro CD release characteristics of the three dosage forms. It is currently widely accepted that there is no interaction between CD and LD during the absorption process in the intestines [19,23]. Levodopa has a narrow absorption window in the proximal small intestine (duodenum and jejunum). It is actively transported across the intestinal epithelium via large neutral amino acid transporters (LAT1s). Specifically, the transporter system SLC7A9–SLC3A1 was recently shown to mediate intestinal LD transport, with leucine and arginine acting as competitors. Transport into the central nervous system, however, is mediated by LAT transporter SLC6A19 [19]. Carbidopa, in contrast, is poorly absorbed and does not rely on LAT1 or similar amino acid transporters [23]. Its absorption is thought to occur via passive diffusion or other non-saturable mechanisms. Our results challenge these notions. Given that all three dosage forms contain the same amount of CD and their in vitro release profiles are highly comparable, why should CD blood levels of LXM.5-1 and LXM.5-2 be higher than those of Sinemet^®^, assuming there is no interaction between CD and LD absorption? The model chosen might offer a potential explanation. The uptake of the two molecules may differ between pigs and humans. However, despite pigs often being used as a model system, there have been no reports on this so far. Additionally, it has to be at least considered that this study used anesthetized piglets fasted for at least 12 h to ensure an empty stomach. While both conditions might impact the transport of the dosage forms and the absorption of CD and LD slightly, this setup was chosen due to several reasons: Primarily, gastro endoscopic application is the only viable method to ensure the structural integrity of the 3D screen-printed dosage form, which was the main variable tested in this study. Secondly, an empty stomach is the closest option to standardized conditions in terms of the pH and the placement of the dosage forms inside the stomach. Additionally, the commercially available Sinemet^®^ is advised to not be taken together with meals which are rich in proteins, as this can delay the absorption [22]. As a consequence, if the stomachs of the piglets were filled with various amounts of protein-rich feed, this might impact the absorption rate and speed and therefore disallow comparison between piglets.

On the other hand, our current knowledge of the absorption of CD and LD in humans is certainly limited by the fact that, until now, it has not been possible to administer them sequentially via the oral route. In fact, studies demonstrating that CD augmented the bioavailability were performed in a hybrid fashion: LD was given intravenously, while CD was administered orally [10,18,24]. Although this setting was suitable for observing and assessing the effect of CD on LD bioavailability, it could not address the question of whether the two interact in the intestinal absorption process. Therefore, our data could provide the first experimental evidence of such an interaction. Since the difference between Sinemet^®^ and LXM.5-1 and LXM.5-2 lies in the initial amount of LD released (as assessed by the ratio of both drugs, see Figure 3), it is theoretically possible that the absorption of both molecules occurs, at least in part, via overlapping mechanisms.

Another theoretical assumption is that the potential competition for uptake mechanisms could impact LD blood levels directly. Specifically, once all the CD has been cleared from the intestine, all the “shared” channels will be available for LD uptake. This could explain the higher peak LD blood levels observed for both LXM.5-1 and LXM.5-2 compared with Sinemet^®^. As an alternative explanation, the increase in LD peak levels could be solely due to the stronger inhibition of LD degradation based on higher CD blood levels. The LD shoulders seen with LXM.5-1 and LXM.5-2 are likely dependent on several factors: the height of the LD peak, the sustained CD blood levels that reflect LD degradation inhibition capacity, the tailoring of the LD release to the upper intestine (LXM.5-2) through the addition of pH-sensitive polymers to the LD compartment, and the sequential release interval. Additional studies will be needed to determine the specific role of these factors. The current study suggests that the longer interval observed in LXM.5-2 (compared with LXM.5-1) and the boost of LD release enabled and mediated by pH-sensitive polymers both contribute to higher peak levels and a greater shoulder. Further studies will address this by varying (i) the length of the sequential release interval, (ii) the pH-sensitive polymers, (iii) the tablet architecture to gradually increase pH-guided LD release (which can be achieved by adding further layers with pH-sensitive polymers).

The 3D screen-printed novel oral CD/LD dosage forms, LXM.5-1 and LXM.5-2, and their potential variations, could transform the pharmacotherapy of Parkinson’s disease. Their prolonged and smooth LD blood levels would avoid pulsatile dopaminergic stimulation which is correlated with motor complications notably motor fluctuations and dyskinesias [9]. Compared with intestinal pump systems, which have been shown to effectively reduce dyskinesias and motor fluctuations by maintaining continuous LD blood levels [25], LXM.5-1 and LXM.5-2 can be easily administered to patients at all stages of the disease. It is tempting to speculate whether this therapy could slow down the progression of the disease itself. Assuming that LXM.5-1 and LXM.5-2 would positively impact the symptoms of the disease—which is highly likely, given the achieved LD blood levels—their use would directly impact patients’ lives. Based on the sustained blood levels, the expected number of daily intakes would be significantly reduced to about 2-3 doses per day. This is life-changing, particularly in the later stages of the disease when patients require 6-8 doses per day, not to mention the fact that they must be taken on an empty stomach.

The safety profile of LXM.5-1 and LXM.5-2 is expected to be comparable to existing LD products. This is because LD peak levels fall well within the range of marketed products. Peak levels of both novel dosage forms were only 30–50% higher than those of 25/100 Sinemet^®^ (which comes at strengths of 25/100 and 50/200), whereas bioavailability was between 211.36% and 383.64%. The situation for CD needs further consideration, as the new dosage forms appear to triple its peak blood levels. Further experiments will be required to determine the amount of CD necessary to increase LD bioavailability.

Finally, after more than 50 years of LD therapy, LXM.5-1 and LXM.5-2, along with their variations, offer the potential for a more personalized approach to Parkinson’s disease treatment. The flexibility of the 3D screen printing technology allows for variations in the content of CD and LD, as well as in the interval between their release. By taking sequential blood level measurements, the most appropriate dosage form could be selected for an individual patient, based on its drug content and release interval.

## 5. Conclusions

In conclusion, 3D screen printing enables the compartmentalization of oral dosage forms. Compartments can contain different drugs. They may also have different functional characteristics. Here, we demonstrate the potential of this technology by creating oral dosage forms that release CD and LD sequentially from distinct compartments and tailor LD release to the upper intestine by integrating pH-sensitive polymers. The resulting product candidates surpass marketed LD formulations in terms of both bioavailability and the duration of LD blood levels. For the first time, they offer relatively continuous dopaminergic stimulation from an oral dosage form. Therefore, these candidates and their variations have the potential to transform the pharmacotherapy of Parkinson’s disease.

## 6. Patents

The use of 3D screen printing technology for the production of drug delivery systems as well as the controlled, sequential release of one or more and different APIs (including carbidopa and levodopa) described here is legally protected by numerous patents (US2023/0078273 A1, WO2019/129360 A1, US2016121599, WO2020127388, etc.).

## Figures and Tables

**Table 1:** The compositions of carbidopa and levodopa pastes (P1 and P2, respectively) of LXM.5-1 are displayed below. Since the two pastes have highly comparable compositions, LXM.5-1′s geometry essentially controls drug release.

(a) Paste P1						(b) Paste P2					
**No.**	**Ingredients**	**Function**	**wt% Paste**	**wt% Tablet**	**mg per Tablet**	**No.**	**Ingredients**	**Function**	**wt% Paste**	**wt% Tablet**	**mg per Tablet**
**1**	Carbidopa	API	23.33	50	25	**1**	Levodopa	API	33.26	60.47	100
**2**	Klucel LF Pharma	Binder	2.36	5.07	2.533	**2**	Klucel LF Pharma	Binder	2.2	4.01	6.625
**3**	Avicel PH 105	Filler	14.45	30.97	15.486	**3**	Avicel PH 105	Filler	13.47	24.49	40.5
**4**	Starch 1500	Filler	2.05	4.4	2.199	**4**	Starch 1500	Filler	1.91	3.48	5.75
**5**	Glycerol	Humectant	3.12	6.69	3.346	**5**	Glycerol	Humectant	2.91	5.29	8.75
**6**	Talc	Anti-Tacking Agent	0.89	1.91	0.956	**6**	Talc	Anti-Tacking Agent	0.83	1.51	2.5
**7**	Silfar 350	Anti-Foaming Agent	0.45	0.96	0.478	**7**	Silfar 350	Anti-Foaming Agent	0.42	0.76	1.25
**8**	L-Ascorbic acid	Anti-Oxidation Agent	0.12	0.25	0.124	**8**	L- (+) -Ascorbic acid	Anti-Oxidation Agent	0.11	0.2	0.325
**9**	Citric acid	pH-Agent	1.01	2.16	1.083	**9**	Water	Solvent	44.88	-	-
**10**	Water	Solvent	52.22	-	-						
	Total		100	100	51.205		Total		100	100	165.708

**Table 2:** The compositions of the Carbidopa- (a; P3) and Levodopa-containing pastes (b; P4) that make up LXM.5-2.

(a) Paste P3						(b) Paste P4					
**No.**	**Ingredients**	**Function**	**wt% Paste**	**wt% Tablet**	**mg per Tablet**	**No.**	**Ingredients**	**Function**	**wt% Paste**	**wt% Tablet**	**mg per Tablet**
**1**	Carbidopa	API	20.62	45.27	25	**1**	Levodopa	API	27.6	60.15	100
**2**	Klucel LF Pharma	Binder	1.88	4.12	2.277	**2**	Klucel LF Pharma	Binder	1.1	2.41	4
**3**	Ac-Di-Sol	Disintegrant	3.46	7.6	4.197	**3**	Shin-Etsu AQOAT AS-LF	Enteric Polymer	7.29	15.88	26.4
**4**	Mannogem Emerald	Filler	12.11	26.6	14.688	**4**	Ammonia 25 %	pH-Agent	0.74	1.6	2.664
**5**	RxCIPIENTS^®^ FM1000	Disintegrant Agent	2.37	5.21	2.878	**5**	Avicel PH-105	Filler	4.86	10.59	17.6
**6**	Talc	Anti-Tacking Agent	0.62	1.37	0.755	**6**	Talc	Anti-Tacking Agent	0.88	1.92	3.2
**7**	Silfar 350	Anti-Foaming Agent	0.21	0.46	0.252	**7**	Glycerol	Humectant	1.55	3.37	5.6
**8**	Miglyol 812 N	Lubricant	1.01	2.21	1.223	**8**	Triethylcitrate	Plasticizer	0.66	1.44	2.4
**9**	Glycerol	Humectant	2.2	4.82	2.662	**9**	Silfar SE 4	Anti-Foaming Agent	1.1	2.41	4
**10**	L- (+) -Ascorbic acid	Anti-Oxidation Agent	0.12	0.27	0.15	**10**	L- (+) -Ascorbic acid	Anti-Oxidation Agent	0.11	0.24	0.4
**11**	Citric acid	pH-Agent	0.94	2.06	1.139	**11**	Water	Solvent	54.1	-	-
**12**	Water	Solvent	54.46	-	-						
	Total		100	100	55.221		Total		100	100	166.264

**Table 3:** Physicochemical characteristics of 3D screen-printed LXM.5-1 and LXM.5-2.

Parameter	Unit	Target	LXM.5-1	LXM.5-2
Hardness	[N]		69.89 ± 2.75	164.80 ± 5.10
Friability	[%]		0.0326	0.0960
Mass uniformity	m [g]		212.58 ± 3.68	256.65 ± 1.55
Dimensions	h [mm]	3.6	3.38 ± 0.09	3.73 ± 0.01
	d [mm]	14	13.87 ± 0.05	13.96 ± 0.01
	b [mm]	5	5.07 ± 0.04	5.18 ± 0.02
Dissolution	100% CD release		after 60 min	after 30 min
	100% LD release		after 120 min	after 195 min
Residual moisture	gravimetric		6.98%	-
	Karl Fischer titration		-	2.42% (m/m)

Tablets were subjected to indicated analyses applying respective methods. Results for hardness and mass uniformity are shown as mean ± standard deviation.

**Table 4:** The subsequent table presents a comparison of the areas under the curve of the marketed product Sinemet^®^ with LXM.5-1 and LxM.5-2. Furthermore, the relative bioavailability of LXM.5-1 and LXM.5-2 related to Sinemet^®^ was calculated. It is essential to acknowledge that values are derived from different experiments, thus necessitating their differentiation to ensure a comprehensive analysis. Statistical analysis by Anova demonstrated AUCs for LD (but not for CD) to differ significantly for LXM.5-1 and Sinemet: *p* = 0.0019, LXM.5-2 and Sinemet: *p* = 0.0018, as well as LXM.5-1 and LXM.5-2: *p* = 0.0053.

Sample		AUC [µg × min/mL × kg]	Relative Bioavailability [%]
Sinemet^®^	Carbidopa	5.04 ± 0.03	100
	Levodopa	11.80 ± 0.02	100
LXM.5-1	Carbidopa	15.56 ± 0.83	308.73
	Levodopa	24.94 ± 1.11	211.36
LXM.5-2	Carbidopa	15.81 ± 0.55	313.69
	Levodopa	45.27 ± 4.46	383.64

**Figure A2:**
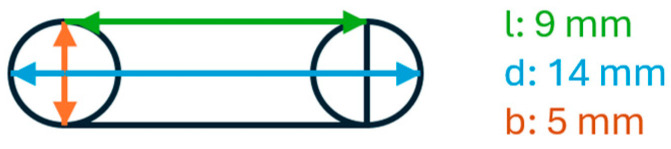
Schematic layout (top view) of the tablets LXM.5-1 and LXM.5-2. The length (l) of the tablet is 9 mm, the diameter (d) is 14 mm, and the broad (b) is 5 mm. The height is not displayed in the top view, but was 3.6 mm.

## Data Availability

The data presented in this study are available on request from the corresponding author.

## Abbreviations

The following abbreviations are used in this manuscript:

API	Active pharmaceutical ingredient
3D-SP-tablet	3D screen-printed tablet
PD	Parkinson’s Disease
CD	Carbidopa
LD, L-Dopa	Levodopa
Ph. Eur.	European Pharmacopeia
USP	United States Pharmacopeia
bw	Body weight
Tab	Tablet
SA/V	Surface area to volume ratio
CDS	Continuous dopaminergic stimulation
LCIG	Levodopa–carbidopa intestinal gel

## Appendix A

**Table A1:** The theoretical dimensions of the tablet LXM.5-1 are delineated in the table below. The proportion of the surface area to volume ratio of the compartments is opposite to the tablet LXM.5-2.

	l [mm]	h [mm]	b [mm]	SA [mm^2^]	V [mm^3^]	SA/V [mm^−1^]	SA/V [mm^−1^]	SA Layer/Total SA [%]
CD	14	0.44	5	79.4674	28.4394	2.7943	0.3415	
LD	14	2.72	5	91.6912	175.8072	0.5215	0.3941	36.58
CD	14	0.44	5	79.4674	28.4394	2.7943	0.3415	63.42
Total	14	3.6	5	250.626	232.686	1.0771	1.0771	100

**Table A2:** The determined dimensions of tablet LXM.5-2 are shown in the table below.

	l [mm]	h [mm]	b [mm]	SA [mm^2^]	V [mm^3^]	SA/V [mm^−1^]	SA/V [mm^−1^]	SA Layer/Total SA [%]
LD	14	2.72	5	156.3262	175.8072	0.8892	0.6718	62.37
CD	14	0.88	5	94.2998	56.8788	1.6579	0.4053	37.63
Total	14	3.6	5	250.626	232.686	1.0771	1.0771	100
